# SENP2 Promotes VSMC Phenotypic Switching via Myocardin De-SUMOylation

**DOI:** 10.3390/ijms232012637

**Published:** 2022-10-20

**Authors:** Min Liang, Zhaohua Cai, Yangjing Jiang, Huanhuan Huo, Linghong Shen, Ben He

**Affiliations:** Heart Center, Shanghai Chest Hospital, Shanghai Jiao Tong University, 241 West Huaihai Road, Shanghai 200030, China

**Keywords:** SENP2, SUMOylation, myocardin, VSMC, phenotypic switching

## Abstract

Myocardin is a master regulator of smooth muscle cell (SMC) differentiation, which induces the expression of smooth-muscle-specific genes through its direct association with serum response factor (SRF). During the past two decades, significant insights have been obtained regarding the regulatory control of myocardin expression and transcriptional activity at the transcriptional, post-transcriptional, and post-translational levels. However, whether and how SUMOylation plays important roles in modulating myocardin function remain elusive. In this study, we found that myocardin is modified by SUMO-1 at lysine 573, which can be reversibly de-conjugated by SENP2. SUMO-1 modification promotes myocardin protein stability, whereas SENP2 facilitates its proteasome-dependent degradation. Moreover, we found that PIAS4 is the SUMO E3 ligase that enhances the SUMOylation and protein stability of myocardin. Most importantly, we found that SENP2 promotes phenotypic switching of VSMC. We therefore concluded that SENP2 promotes VSMC phenotypic switching via de-SUMOylation of myocardin and regulation of its protein stability.

## 1. Introduction

Vascular smooth muscle cells (VSMCs) are a highly differentiated cell type, yet retaining remarkable phenotypic plasticity. This plasticity has been frequently associated with phenotypic switching from a contractile to a synthetic phenotype and plays a central role in numerous vascular diseases. Myocardin is a well-known transcriptional coactivator of serum response factor (SRF) responsible for the maintenance of the VSMC contractile phenotype [1]. Binding of myocardin to SRF on the CArG box-containing target genes transcriptionally activates smooth-muscle-specific markers including α-smooth muscle actin (α-SMA, *Acta2*), smooth muscle 22α (SM22α, *Tagln*), and smooth muscle myosin heavy chain (SM-MHC, *Myh11*). Therefore, disruption of myocardin function has been strongly implicated in a wide variety of vascular diseases, including atherosclerosis, hypertension, and aortic aneurysm [2,3,4,5,6,7].

The expression and transcriptional activity of myocardin are widely regulated at the transcriptional, post-transcriptional, and post-translational levels. Posttranslational modifications (PTMs), such as phosphorylation, acetylation, ubiquitination, and SUMOylation, are important regulators of cell signaling, due to the transient and often reversible nature of these modifications. It has been well demonstrated that myocardin can be regulated by PTMs including phosphorylation, acetylation, and SUMOylation [8,9,10,11]. MAPK p44/42 phosphorylates myocardin and, subsequently, increases its transcriptional activity [8]. However, phosphorylation of myocardin by GSK-3β inhibits its transcriptional activity in cardiomyocytes [9]. Moreover, myocardin acetylation mediated by p300 is required for the binding between myocardin and srf and the transcriptional activity of myocardin [10].

SUMOylation is an essential PTM that covalently conjugates small ubiquitin-like modifier (SUMO) to target proteins and regulates the interactome, protein stability, subcellular localization, and transcriptional activity of its target proteins [12,13]. Myocardin SUMOylation mediated by SUMO-1/PIAS1 has been demonstrated to increase its transcriptional activity [11], yet whether SUMO modification affects the protein stability of myocardin remains unknown. Sentrin-/SUMO-specific proteases (SENPs) de-SUMOylate modified proteins and play critical roles in maintaining the balance of SUMOylation/de-SUMOylation. We previously demonstrated that SENP3, a redox-sensitive SUMO2/3 protease, plays important roles in VSMC function and vascular remodeling [14]. However, the role of other SENPs in VSMC function has never been investigated.

In the present study, we examined the SUMO modification of myocardin and demonstrated that myocardin is modified by SUMO-1 at lysine 573, which can be reversibly de-conjugated by SENP2. SUMOylation promotes myocardin stability, whereas SENP2 facilitates its proteasome-dependent degradation and induces VSMC phenotypic switching. These findings suggest that SENP2 is an important regulator of VSMC phenotype via myocardin de-SUMOylation.

## 2. Results

### 2.1. Myocardin Is SUMOylated at Lysine 573, Which Can Be De-SUMOylated by SENP2

**Figure 1:**
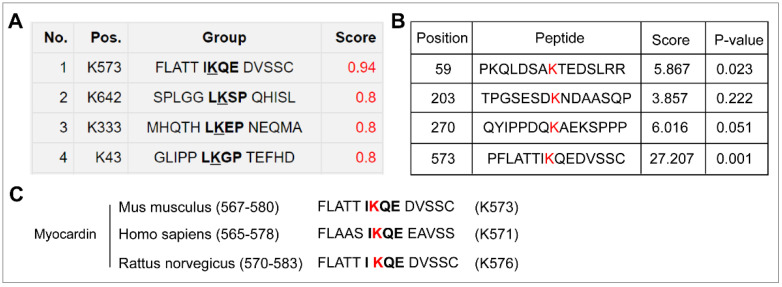
The prediction and conservation of SUMOylation sites in myocardin. (**A**,**B**) The putative SUMOylation sites in myocardin isoform B (Mus musculus) were predicted by the SUMOplot^TM^ Analysis Program online tool (https://www.abcepta.com/sumoplot, accessed on 18 February 2022) (**A**) and SUMOsp2.0 software (**B**). One lysine residue (lysine 573) was consistent in the results. (**C**) Alignment of orthologous myocardin amino acid sequences in *Mus musculus*, *Homo sapiens*, and *Rattus norvegicus* indicates evolutionary conservation of SUMOylation sites in myocardin. (**A**–**C**), The canonical SUMO-binding motif is defined as ψKX(D/E), where ψ represents an aliphatic or hydrophobic amino acid residue, ‘K’ in the consensus sequence is the SUMO acceptor lysine, and ‘X’ is any amino acid, which is adjacent to an acidic residue (Asp/Glu).

**Figure 2:**
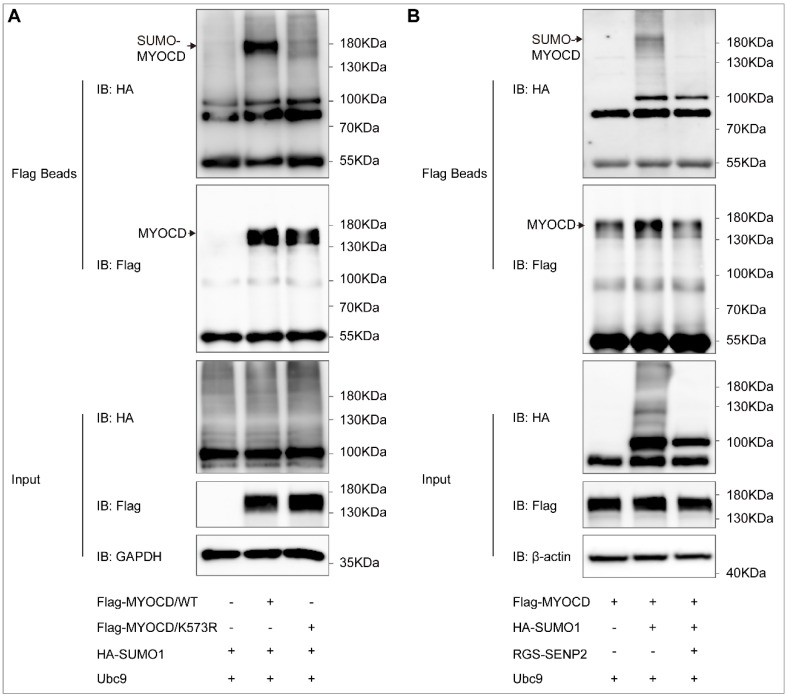
Myocardin is SUMOylated at lysine 573, which can be de-SUMOylated by SENP2. (**A**) HEK-293T cells were transfected with Flag-myocardin/WT (Flag-MYOCD/WT) or Flag-myocardin/K573R (Flag-MYOCD/K573R), HA-SUMO1, and Ubc-9 for 24 h. The SUMOylation of Flag-MYOCD was determined by the IP assay using Flag beads and Western blotting using anti-Flag, anti-HA, and anti-GAPDH antibodies. (**B**) HEK-293T cells were transfected with Flag-myocardin (Flag-MYOCD), HA-SUMO1, RGS-SENP2, and Ubc-9 for 24 h. The SUMOylation of Flag-MYOCD was determined by the IP assay using Flag beads and Western blotting using anti-Flag, anti-HA, and anti-β-actin antibodies.

To investigate whether myocardin can be SUMOylated, we first predicted the SUMOylation possibility of myocardin using computational-system-based software including SUMOsp 2.0 and SUMOplot^TM^. One lysine residue (lysine 573) for myocardin isoform B (Mus musculus) was consistent in the results of SUMOplot^TM^ (Figure 1A) and SUMOsp 2.0 (Figure 1B) and highly conserved among different species (Figure 1C). Meanwhile, myocardin SUMOylation was detected by immunoprecipitation in HEK-293T cells co-expressed with Flag-myocardin and HA-SUMO-1. Using the Flag beads pull-down assay, SUMOylation of myocardin was demonstrated (Figure 2A). The result showed that myocardin can be mono-SUMOylated, while the SUMOylation was abolished in a site-directed mutant in which lysine 573 was converted to arginine (myocardin-K573R mutant) (Figure 2A). Moreover, we found that SENP2 de-conjugated SUMO-1 from myocardin (Figure 2B). These results suggest that myocardin is modified by SUMO-1 at the conserved lysine 573, which can be reversibly de-conjugated by SENP2.

### 2.2. SUMO-1 Promotes Myocardin Stability, Whereas SENP2 Facilitates Its Proteasome-Dependent Degradation

**Figure 3:**
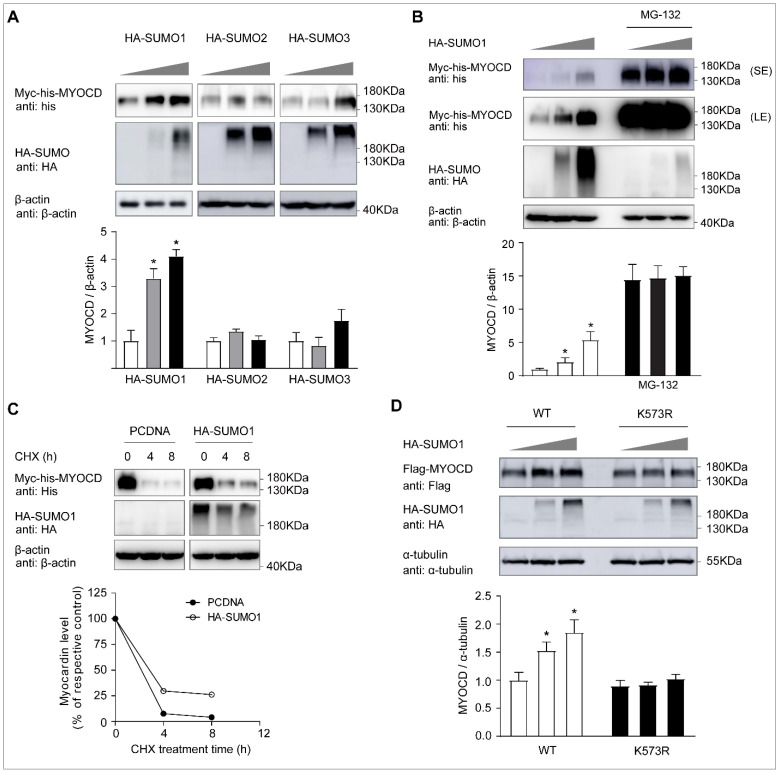
SUMO-1 promotes the protein stability of myocardin. (**A**) HEK-293T cells were transfected with Myc-his-myocardin (Myc-his-MYOCD) and increasing amounts of HA-SUMO1, HA-SUMO2, or HA-SUMO3 for 24 h. The levels of Myc-his-MYOCD in whole-cell lysates were determined by Western blotting with anti-his, anti-HA, and anti-β-actin antibodies (n = 3, * *p* < 0.05). (**B**) HEK-293T cells were transfected with Myc-his-MYOCD and increasing amounts of HA-SUMO1 for 24 h, in the presence or absence of MG132 (10 μM) for the last 10 h. Lysates were prepared and analyzed by Western blotting (n = 3, * *p* < 0.05). SE, short-time exposure; LE, long-time exposure. (**C**) HEK-293T cells were transfected with Myc-his-MYOCD and PCDNA or HA-SUMO1 for 24 h and were subsequently exposed to the protein synthesis inhibitor cycloheximide (CHX) for the indicated time. Lysates were prepared and analyzed by Western blotting. The relative level of Myc-his-MYOCD was evaluated by densitometry and normalized to β-actin. (**D**) HEK-293T cells were transfected with Flag-myocardin/WT (Flag-MYOCD/WT) or Flag-myocardin/K573R (Flag-MYOCD/ K573R) and increasing amounts of HA-SUMO1 for 24 h. The levels of Flag-MYOCD in whole-cell lysates were determined by Western blotting with anti-Flag, anti-HA, and anti-α-tubulin antibodies (n = 3, * *p* < 0.05).

As SUMOylation modulates the protein stability, subcellular localization, and transcriptional activity of its target proteins [12,13], here, we investigated the protein level change of myocardin after SUMO modification. The protein level of myocardin was dose-dependently increased by SUMO-1 overexpression, whereas there was no significant change of the myocardin protein level after SUMO-2 and SUMO-3 overexpression (Figure 3A). Moreover, SUMO-1-mediated myocardin upregulation was abolished following treatment with the 26S proteasome inhibitor MG-132 (Figure 3B).

To further explore the role of SUMOylation in modulating myocardin stability, we examined the myocardin half-life in HEK-293T cells treated with the protein synthesis inhibitor cycloheximide (CHX). Co-expression of SUMO-1 with myocardin increased the half-life of myocardin (Figure 3C). In addition, the increased protein level of myocardin after SUMO-1 overexpression was abolished in the myocardin-K573R mutant form (Figure 3D), suggesting that SUMO modulation at lysine 573 promotes the protein stability of myocardin.

**Figure 4:**
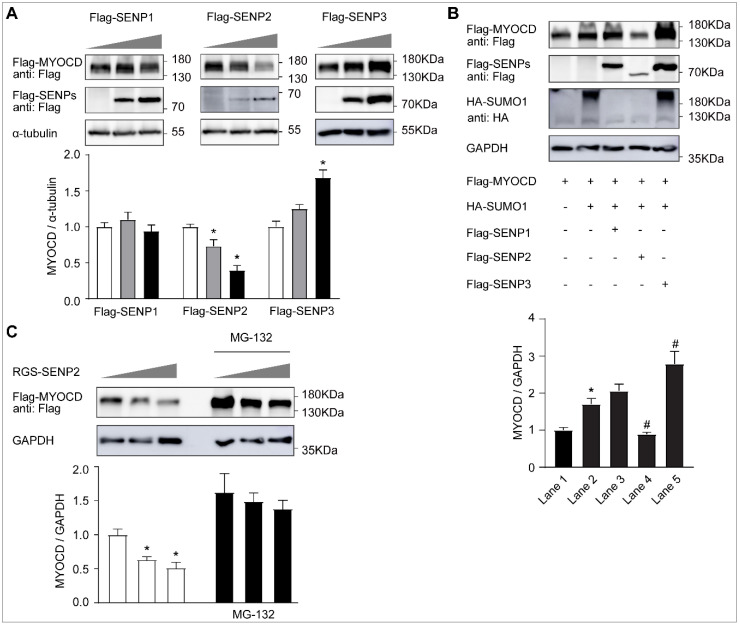
SENP2 promotes proteasome-dependent degradation of myocardin. (**A**) HEK-293T cells were transfected with Flag-myocardin (Flag-MYOCD) and increasing amounts of Flag-SENP1, Flag-SENP2, or Flag-SENP3 for 24 h. The levels of Flag-MYOCD in whole-cell lysates were determined by Western blotting with anti-Flag and anti-α-tubulin antibodies (n = 3, * *p* < 0.05). (**B**) HEK-293T cells were transfected with Flag-MYOCD, HA-SUMO1, and Flag-SENP1, Flag-SENP2, or Flag-SENP3 for 24 h. Lysates were prepared and analyzed by Western blotting (n = 3, * *p* < 0.05 vs. Lane 1, ^#^ *p* < 0.05 vs. Lane 2). (**C**) HEK-293T cells were transfected with Flag-MYOCD and increasing amounts of RGS-SENP2 for 24 h, in the presence or absence of MG132 (10 μM) for the last 10 h. Lysates were prepared and analyzed by Western blotting (n = 3, * *p* < 0.05).

In contrast, we found that the protein level of myocardin was dose-dependently decreased by SENP2 overexpression (Figure 4A). However, there was no change of the myocardin protein level after SENP1 overexpression, whereas it seems that the protein level of myocardin was increased after SENP3 overexpression (Figure 4A). This was further confirmed by the finding that only SENP2 can specifically reverse the increased protein expression of myocardin after SUMO-1 overexpression (Figure 4B). Moreover, SENP2-mediated myocardin elimination was abolished by proteasome inhibitor MG-132 (Figure 4C).

Taken together, these results suggest that SUMO-1 promotes the protein stability of myocardin, whereas SENP2 facilitates its proteasome-dependent degradation.

### 2.3. PIAS4 Is the SUMO E3 Ligase That Mediates Myocardin SUMOylation

**Figure 5:**
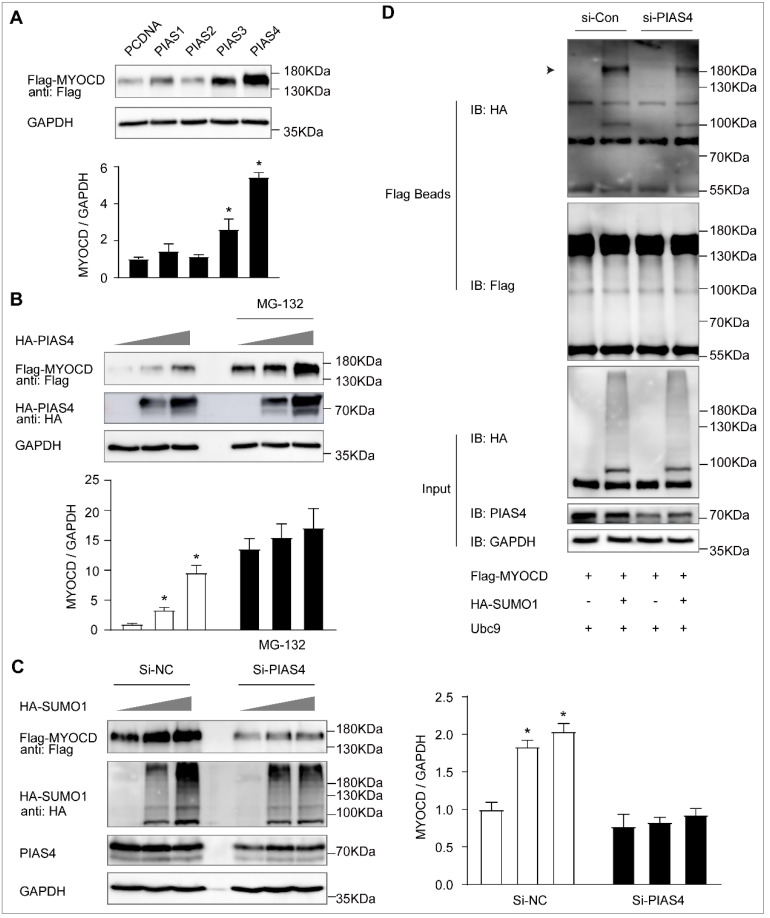
PIAS4 is the SUMO E3 ligase that mediates myocardin SUMOylation. (**A**) HEK-293T cells were transfected with Flag-MYOCD and HA-PIAS1, HA-PIAS2, HA-PIAS3, or HA-PIAS4 for 24 h. Lysates were prepared and analyzed by Western blotting (n = 3, * *p* < 0.05). (**B**) HEK-293T cells were transfected with Flag-MYOCD and increasing amounts of HA-PIAS4 for 24 h, in the presence or absence of MG132 (10 μM) for the last 10 h. Lysates were prepared and analyzed by Western blotting. (**C**) HEK-293T cells were transfected with control siRNA (si-NC) and PIAS4 siRNA (si-PIAS4) for 48 h and then transfected with increasing amounts of HA-SUMO1 for another 24 h. Lysates were prepared and analyzed by Western blotting (n = 3, * *p* < 0.05). (**D**) HEK-293T cells were transfected with control siRNA (si-NC) and PIAS4 siRNA (si-PIAS4) for 48 h and then transfected with or without HA-SUMO1 for another 24 h. The SUMOylation of Flag-MYOCD was determined by the IP assay using Flag beads and Western blotting using anti-Flag, anti-HA, anti-PIAS4, and anti-GAPDH antibodies (n = 3, * *p* < 0.05).

The protein inhibitor of the activated STAT (PIAS) family, which includes PIAS1, PIAS2 (PIASx), PIAS3, and PIAS4 (PIASy), is the well-known SUMO E3 ligase that facilitates the SUMO modification of target proteins [15]. Among the PIAS family, overexpression of PIAS4 dramatically increased the protein level of myocardin (Figure 5A), which is consistent with the data after SUMO-1 overexpression. This was further confirmed by the result that the protein level of myocardin was dose-dependently increased by PIAS4 overexpression (Figure 5B). Furthermore, we found that SUMO-1-mediated myocardin upregulation was completely abolished after PIAS4 knockdown (Figure 5C). Most importantly, SUMO-1 modification of myocardin was dramatically decreased after PIAS4 knockdown (Figure 5D). Taken together, these results indicate that PIAS4 is the SUMO E3 ligase that is required for myocardin SUMOylation and promotes its protein stability.

### 2.4. SENP2 Promotes Phenotypic Switching of VSMC In Vitro

**Figure 6:**
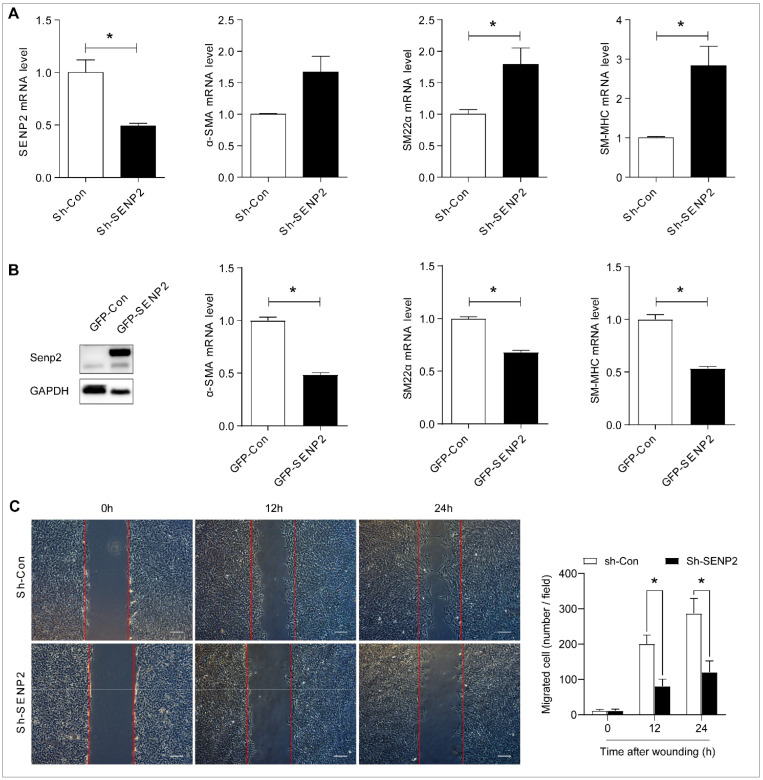
SENP2 promotes VSMC phenotypic switching in vitro. (**A**) VSMCs were infected with control lentivirus (Sh-Con) and sh-SENP2 lentivirus (Sh-SENP2) for 72 h. The expression of SENP2, α-SMA, SM22α, and SM-MHC was examined by real-time quantitative PCR (RT-qPCR). (**B**) VSMCs were infected with adenovirus containing GFP-Con or GFP-SENP2 for 48 h. The expression of SENP2 was examined by Western blotting. The expression of α-SMA, SM22α, and SM-MHC was examined by RT-qPCR. (**C**) VSMCs were infected with control lentivirus (Sh-Con) and sh-SENP2 lentivirus (Sh-SENP2) for 48 h. Monolayer confluent cells were serum-starved overnight and scraped in the presence of normal growth medium to stimulate VSMC migration toward the wound area. Representative images of the in vitro scratch-wound assay and quantification of migrated cells are presented. Scale bars: 200 µm. (**A**–**C**), n = 3, * *p* < 0.05.

To investigate the role of SENP2 in phenotypic switching of VSMCs, we first performed the real-time quantitative PCR assay after SENP2 knockdown or overexpression and analyzed the expression of VSMC-specific genes, including α-SMA, SM-22α, and SM-MHC. The expression of these genes was significantly upregulated in VSMCs infected with sh-SENP2 lentivirus, compared with VSMCs infected with sh-Con lentivirus (Figure 6A). Accordingly, when VSMCs were infected with adenovirus containing GFP-SENP2 to overexpress SENP2, the expression of VSMC-specific genes was significantly downregulated (Figure 6B). Moreover, under normal growth conditions, VSMCs infected with sh-SENP2 lentivirus exhibited significantly decreased cell migration compared with VSMCs infected with sh-Con lentivirus (Figure 6C). These results suggest that SENP2 promotes phenotypic switching of VSMCs.

## 3. Discussion

**Figure 7:**
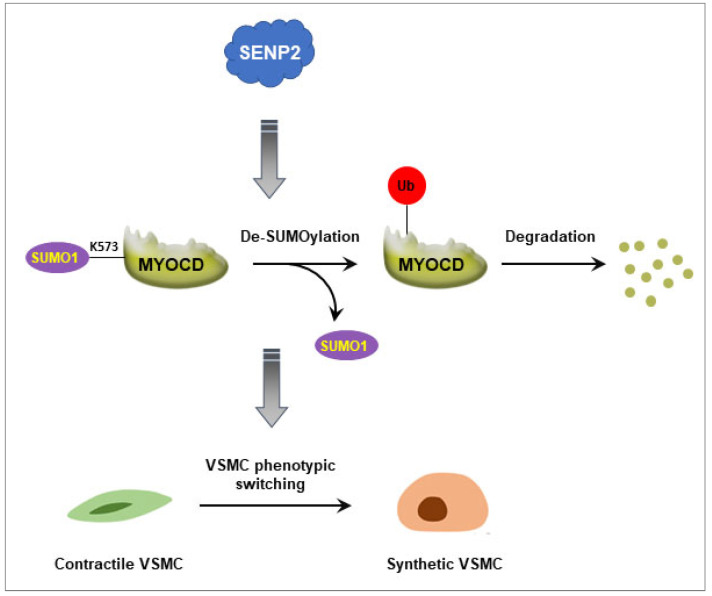
Schematic illustrating how SENP2 promotes VSMC phenotypic switching. In this study, we propose that SENP2 promotes VSMC phenotypic switching via de-SUMOylation of myocardin and regulation of its protein stability.

SENPs are cysteine proteases that play critical roles in maintaining the SUMO/de-SUMOylation balance of modified proteins required for normal cellular physiology. Six isoforms of SENPs have been identified in humans (SENP1, SENP2, SENP3, SENP5, SENP6, and SENP7) [16]. While previous studies have mainly focused on the pivotal role of SENPs in the development of cancer [17,18,19,20,21,22], in recent years, the role of SENPs and SUMOylation in vascular diseases has attracted considerable attention [14,23,24,25,26,27]. SENP1 modulates the SUMOylation of GATA2 and leads to endothelial dysfunction in graft atherosclerosis [23]. SENP2 regulates the SUMOylation of ERK5 and P53 and plays a critical role in disturbed flow-induced endothelial dysfunction and atherosclerosis [24,25]. In our previous studies, we demonstrated that SENP3 mediates vascular remodeling via de-SUMOylation of β-catenin [14]. In the present study, we found that SENP2 promotes VSMC phenotypic switching via de-SUMOylation of myocardin and regulation of its protein stability (Figure 7).

Myocardin is a critical regulator of smooth-muscle-specific genes through its direct association with SRF. The expression and transcriptional activity of myocardin have been studied to be widely regulated at the transcriptional, post-transcriptional, and post-translational levels. Myocardin is subjected to multiple PTMs, which mainly modulates its transcriptional activity [8,9,10,11]. However, little is known about the regulation of its protein stability through PTMs, especially SUMOylation. In the present study, we found that myocardin is modified by SUMO-1, which can be reversibly de-conjugated by SENP2. Moreover, SENP2 facilitates proteasome-dependent degradation of myocardin, whereas SUMOylation promotes its protein stability. It is well established that SUMOylation can serve as a targeting signal recognized by SUMO-targeted ubiquitin ligases (STUbLs) [28]. STUbLs are recruited to SUMOylated proteins and catalyze their ubiquitination and degradation [29,30,31]. Our previous results indicated that β-catenin is targeted to the ubiquitin-proteasome system for degradation in a SUMOylation-dependent manner [14]. However, many studies have demonstrated that SUMOylation enables its target proteins to be more stable by inhibiting the ubiquitin-proteasome system. For example, SUMOylation of the MCL1 protein promotes its stability by inhibiting the ubiquitin-proteasome pathway [32]. In the present study, we found that SUMOylation of the myocardin protein facilitates its protein stability. Therefore, we demonstrate a novel regulatory control of myocardin expression through its SUMO modification.

The PIAS proteins, including PIAS1, PIAS2 (PIASx), PIAS3, and PIAS4 (PIASy), are important SUMO E3 ligases that facilitate the SUMO modification of target proteins and have been strongly implicated in the modulation of the transcriptional activities of various transcription factors [33,34]. Recent findings indicate that PIASs play additional roles in regulating protein stability and signaling transduction pathways [15]. In the present study, we found that PIAS4 is the SUMO E3 ligase that facilitates myocardin SUMOylation and, thus, promotes its protein stability.

## 4. Materials and Methods

### 4.1. Cell Culture

Primary VSMCs were isolated from the thoracic aorta of 6–8-week-old Sprague–Dawley rats using mechanical dissociation as described previously [14,35,36]. VSMCs were cultured in DMEM supplemented with 10% fetal bovine serum (FBS), 100 U/mL penicillin, and 100 μg/mL streptomycin. VSMCs from passages 3–8 were used for in vitro experiments. HEK-293T cells were maintained in DMEM with 10% FBS, 100 U/mL penicillin, and 100 μg/mL streptomycin in an atmosphere containing 5% CO_2_.

### 4.2. Plasmid Constructs and Transfection

Flag-tagged and Myc-his-tagged constructs for wild-type myocardin (Flag-myocardin and Myc-his-myocardin) were generated using standard techniques by cloning the full-length cDNA of myocardin isoform B (Mus musculus) into the pCDNA3.1 (+) −3 × Flag-C and pcDNA3.1-Myc-His (-) A vectors, respectively. The Flag-myocardin lysine to arginine mutant construct (Flag-myocardin/K573R) was generated by site-directed mutagenesis based on the Flag-myocardin construct using a QuikChange Mutagenesis Kit following the described method.

The constructs for HA-PIAS1, HA-PIAS2, HA-PIAS3, and HA-PIAS4 were purchased from Genomeditech (Shanghai, China). The constructs for HA-SUMO-1, HA-SUMO-2, HA-SUMO-3, and Ubc-9 were used in our previous work [14]. Flag-SENP1, Flag-SENP2, and RGS-SENP2 were kindly provided by Prof. Jinke Cheng (Shanghai Jiaotong University School of Medicine, Shanghai, China). The constructs were transiently transfected into cells using Lipofectamine 2000 (Invitrogen, Carlsbad, CA, USA) following the manufacturer’s instructions.

### 4.3. Lentiviral Infection

The lentiviral construct of SENP2 short hairpin RNA (shRNA) (sh-SENP2) was generated by inserting a shRNA oligonucleotide (SENP2 shRNA, 5′-GGTGAATCTCTTCGATCAAGA-3′) into the PGMLV-hU6-MCS-CMV-ZsGreen1-PGK-Puro-WPRE vector (Genomeditech, Shanghai, China). Lentiviral particles containing SENP2 shRNA (sh-SENP2) and the lentivirus-control (sh-Con) were packaged as previously described. For lentiviral transfection, primary VSMCs were plated in 6-well plates and infected with sh-SENP2 and sh-Con lentivirus at 50% confluence in growth media containing polybrene (5 μg/mL). Growth media were refreshed after 24 h, and cells were harvested at 72 h for further experiments.

### 4.4. Adenovirus Infection

Adenovirus containing pADV-mCMV-SENP2-3xFlag-P2A-EGFP (GFP-SENP2) and pADV-mCMV-3xFlag-P2A-EGFP (GFP-Con) was purchased from OBiO technology (Shanghai, China). For adenovirus transfection, primary VSMCs were plated in 6-well plates and infected with AAV-SENP2 and AAV-Con at 70% confluence. Growth media were refreshed at 12 h, and cells were harvested at 48 h for further experiments.

### 4.5. Western Blotting

Whole cell lysates were prepared and quantitated by the BCA assay. Equal amounts of protein per lane were subjected to SDS-PAGE, transferred to a polyvinylidene fluoride (PVDF) membrane (Amersham), and immunoblotted using antibodies against HA (1:4000; Abcam, Burlingame, CA, USA), Flag (1:5000; Sigma-Aldrich, St. Louis, MO, USA), GAPDH (1:1000; Santa Cruz, CA, USA), β-actin (1:1000; Santa Cruz), His-tag (1:1000; Cell Signaling Technology (CST), Danvers, MA, USA), Myc-tag (1:1000; CST), α-Tubulin (1:1000; CST), PIAS4 (1:1000; CST), SENP2 (1:1000; GeneTex, San Antonio, TX, USA), and horseradish peroxidase-conjugated secondary antibodies. Protein bands were detected using enhanced chemiluminescence (Thermo Fisher Scientific, Waltham, MA, USA).

### 4.6. Flag Immunoprecipitation Assay

For the Flag immunoprecipitation assay, transfected cells in a 10 cm dish were lysed in cold Pierce IP lysis buffer (Thermo Fisher, Cat# 87788) supplemented with protease inhibitor cocktail (Roche, Basel, Switzerland; Cat# 11697498001) and 20 mM N-Ethylmaleimide (Sigma, Cat# E3876) on ice for 30 min. The lysates were centrifuged for 15 min at 12,000 rpm. Anti-Flag M2 Affinity Gel (Sigma-Aldrich, Cat# A2220) was added to the cell lysates and incubated at 4 °C overnight. The resin was washed with lysis buffer 5 times. After the last washing, the proteins were eluted in elution buffer and subjected to Western blotting.

### 4.7. RNA Extraction and Real-Time Quantitative PCR 

Total RNA was extracted from the cultured VSMCs using Trizol reagent (Invitrogen), according to the manufacturer’s instructions. Total RNA (1 μg) was reverse transcribed as cDNA utilizing the iScript cDNA Synthesis kit (Bio-rad, Hercules, CA, USA; Cat# 1708881), and RT-qPCR amplification was performed using iQ SYBR Green Supermix (Bio-rad, Cat# 1708882) and the ABI 7300 Real-time system (Applied Biosystems, Carlsbad, CA, USA). The primer sequences used for the detection of α-SMA, SM-22α, and SM-MHC are presented as follows: α-SMA forward: 5′-ATCCGATAGAACACGGCATC-3′; α-SMA reverse: 5′- AGGCATAGAGGGACAGCACA-3′; SM-22α forward: 5′-CGGCAGATCATCAGTTAGAAG-3′; SM-22α reverse: 5′-GGGCTGAGGCTGAGGATAGGT-3′; SM-MHC forward: 5′- ATGCTGGGAAGGTGGACTACAA-3′; SM-MHC reverse: 5′-TGTGCAGGGCTGTGGTTGA-3′. Relative mRNA expression was calculated using the comparative ΔΔCT method, and the resulting values were normalized to GAPDH expression. RT-qPCR was performed in triplicate for each experiment. The results presented represent three independent experiments.

### 4.8. In Vitro Scratch-Wound Assay

For the scratch-wound assay, primary rat VSMCs were seeded into 6-well plates and were infected with sh-SENp2 lentivirus (sh-SENP2) and sh-control (sh-Con). At 48 h after infection, the cells were serum-starved overnight, scraped by sterilized 10 μL pipette tips, washed with PBS to remove the cell debris, and cultured in normal growth medium for an additional 12 and 24 h. Photomicrographs were taken using microscopy. The numbers of cells that migrated into the wound area was quantified.

### 4.9. Statistical Analysis

Values are expressed as the mean ± the standard error of the mean (SEM). Student’s t-test was used for the comparison of two groups. A *p*-value < 0.05 was considered to indicate a statistically significant result.

## 5. Conclusions

This study highlighted the important role of SENP2 in the regulation of myocardin protein stability and VSMC phenotypic switching. The SUMO/de-SUMOylation balance of myocardin mediated by SENP2 and PIAS4 serves as an important modulator of myocardin stability. Our findings suggest that targeting SENP2 or PIAS4 to affect myocardin SUMOylation may thus be a potential therapeutic strategy for VSMC dysfunction and vascular diseases.

## Data Availability

All data supporting this study are contained within the article.
